# Effects of individual versus pair housing during the first 15 d on pre-weaning growth performance and hair cortisol concentrations in dairy calves

**DOI:** 10.5194/aab-69-643-2026

**Published:** 2026-11-09

**Authors:** Şevval Alsaç, Beyzanur Aköz, Erkan Pehlivan

**Affiliations:** 1 Department of Animal Science, Graduate School of Natural and Applied Sciences, Ankara University, Ankara, 06110, Türkiye; 2 Department of Animal Science, Faculty of Agriculture, Ankara University, Ankara, 06110, Türkiye

## Abstract

In modern dairy production systems, calves are separated from their dams shortly after birth and are commonly housed individually, limiting social interactions with peers. Although this management practice aims to reduce the transmission of infectious diseases and to prevent cross-suckling, it may also lead to social isolation. This study investigated the effects of early-life individual versus pair housing of female calves during the first 15 d of the milk-feeding period on pre-weaning growth performance and hair cortisol concentrations (HCCs). A total of 31 Holstein female calves were initially allocated to individual housing ( n=19 ) or pair housing ( n=12 ; six pairs). Of these, 20 calves completed the 85 d follow-up and constituted the primary analysis population (individual housing, n=11 ; pair housing, n=9 ). Calves were housed in zeolite-bedded calf pens for the first 15 d after birth. From day 16 onwards, calves were transferred to a group pen equipped with an automatic milk-feeding robot and remained there until the end of the study (day 85). Body weight (BW) was recorded at birth and at predefined time points, and HCC was measured at birth and on day 85 in a randomly selected subset of 14 calves (7 per treatment). Housing treatment had no significant effect on BW or HCC ( P>0.05 ), whereas the treatment × time interaction was significant for average daily gain (ADG; P<0.05 ). Sensitivity analyses incorporating all available BW and ADG observations supported the primary growth performance findings. However, these findings should be interpreted cautiously because of the small sample size and the difference in space allowance per calf. This study adds evidence regarding individual versus pair housing, limited to the first 15 d before transfer to group housing, showing significantly higher ADG during days 15–30 in calves initially housed individually but no statistical support for the main effects on pre-weaning BW or HCC.

## 1 Introduction

In traditional or natural cattle-rearing systems, calves are raised by their dams and are allowed to establish social interactions with peers or other animals in the herd. In such systems, calves are not separated from their mothers and herd mates, and they are not exposed to major management changes until weaning. In contrast, in modern dairy production systems, calves are commonly moved to individual pens or hutches shortly after birth (Costa et al., 2016), and they are generally kept from suckling their dams and forming social relationships until weaning (Liu et al., 2020).

Individual housing of dairy calves in pens or hutches is widely implemented to reduce the spread of infectious diseases such as diarrhoea and pneumonia and to prevent cross-suckling behaviour (Vasseur et al., 2010). In addition, individual housing facilitates observation and management of calves (Wells et al., 1996). However, individual housing has also been associated with increased fearfulness (Jensen and Larsen, 2014), social isolation (Hötzel et al., 2014), difficulties in adapting to novel environments, and impaired learning ability (Meagher et al., 2015). Recent studies have suggested that raising calves in social housing systems (pair or group housing) may provide advantages over individual housing (Liu et al., 2020; Aghaei et al., 2026). In particular, social housing during the pre-weaning period has been linked to improvements in average daily gain (ADG; Knauer et al., 2021) and solid feed intake (Whalin et al., 2018; McFarland et al., 2024), while well-managed outdoor group housing may support calf health and welfare (Hötzel et al., 2014). Studies comparing calves paired at different ages found no statistical support for an association between pairing age and the occurrence of diarrhoea or bovine respiratory disease (Moroz et al., 2025, 2026). Recent studies have also indicated that early social contact may influence calves' competitive behaviour and the expression of personality traits after subsequent group housing (Suchon et al., 2023; Gingerich et al., 2023). Therefore, evaluations of social housing systems should consider not only production outcomes but also their effects on calf behaviour, cognition, and welfare.

Although multiple indicators can be used to evaluate the effects of housing systems on calf welfare and growth performance, monitoring growth and development remains one of the most widely used approaches. While growth performance is a key indicator of productivity, it may not be sufficient to fully assess animal welfare. Welfare encompasses physical health, opportunities to express natural behaviour, and positive and negative affective states (Mellor et al., 2020). Welfare assessment should therefore include complementary behavioural and physiological indicators. Affective states can be described in terms of valence (positive or negative) and arousal (level of activation). Physiological assessment may include measures of sympathetic–adrenal or hypothalamic–pituitary–adrenal (HPA) activity (Mendl et al., 2010). Because individual housing may expose calves to isolation-related stress, we focused on stress-related physiological activation relevant to the arousal dimension rather than directly assessing emotional valence. Cortisol can be measured in different biological matrices, including blood, saliva, and hair, which reflect different time windows of cortisol exposure. Whereas serum or salivary cortisol generally reflects short-term responses, hair cortisol concentration (HCC) can provide a retrospective indicator of cortisol accumulated during hair growth (Stalder and Kirschbaum, 2012). HCC was therefore selected because hair collection is non-invasive and offers a practical means of assessing cumulative HPA axis activity (Stalder and Kirschbaum, 2012; Heimbürge et al., 2020). However, HCC does not directly assess emotional valence or overall welfare.

Based on the available literature, limited information exists regarding the use of HCC to evaluate the effects of pair housing on stress responses and, consequently, welfare in dairy calves during the pre-weaning period. Furthermore, it remains unclear whether pair housing restricted to the first 15 d of life influences subsequent growth patterns after calves are transferred to group housing. Previous studies have reported improved pre-weaning growth performance in pair-housed calves (Knauer et al., 2021), as well as welfare-related behavioural benefits, including reduced fearfulness towards unfamiliar calves (Jensen and Larsen, 2014). Based on these findings, we hypothesised that pair housing during early life would support improved pre-weaning growth performance and be associated with lower HCC compared with individual housing. Therefore, the aim of this study was to investigate the effects of early-life individual versus pair housing of female dairy calves during the first 15 d of the milk-feeding period on growth performance from birth to weaning and on HCCs.

## 2 Materials and methods

### 2.1 Animals and experimental design

The study initially included 31 female calves born to 2-year-old Holstein heifers raised on a private dairy farm located in İzmir, Türkiye. Calves were randomly allocated to individual housing ( n=19 ) or pair housing ( n=12 ; six pairs). Within the pair-housing treatment, calves born on the same day were paired. A total of 20 calves completed the 85 d follow-up and constituted the primary complete-case analysis population, comprising 11 calves assigned to individual housing and 9 calves assigned to pair housing. The remaining 11 calves (8 assigned to individual housing and 3 assigned to pair housing) died during follow-up due to causes that, based on farm health records and veterinary assessments, were considered to be unrelated to the experimental housing treatments. During the first 15 d after birth, calves in both treatments were housed in zeolite-bedded pens of identical dimensions ( 1.00m×1.95m ). Each individual-housing pen contained one calf, providing 1.95 $m^{2}$ per calf, whereas each pair-housing pen contained two calves, providing approximately 0.98 $m^{2}$ per calf. From day 16 onwards, calves from both treatments were moved to the same group pen, equipped with an automatic milk-feeding robot, and were housed there until the end of the experimental period (day 85). Calves received colostrum for the first 3 d, with 1.5 L provided per meal, three times daily (total: 4.5 $L\;d^{-1}$ ). From day 4 to day 15, calves were fed milk replacer at the same feeding frequency and volume (1.5 L per meal, three times daily; total: 4.5 $L\;d^{-1}$ ). In addition to liquid feeding, calves had ad libitum access to a hay–alfalfa mixed roughage and pelleted calf starter throughout the study (Table 1). After transfer to the group pen, the milk allowance was gradually increased from 6.0 to 8.5 $L\;d^{-1}$ between days 15 and 35, increased further from 8.5 to 10.0 $L\;d^{-1}$ between days 35 and 50, and then gradually decreased from 10.0 to 1.8 $L\;d^{-1}$ from day 50 until weaning at day 85 (Fig. 1). All calves were monitored daily for clinical signs of diarrhoea and respiratory disease throughout the experimental period, and any treatments were recorded according to farm veterinary protocols. Throughout the study, all calves were exposed to the same feeding and climatic conditions.

**Table 1:** Nutrient composition of the milk replacer and calf starter fed during the experimental period.

Nutrient	Milk replacer	Calf starter
Milk powder (%)	50	–
Crude protein (%)	23	22
Crude cellulose (%)	–	8.6
Fat (%)	16	2.7
Crude fibre (%)	0.05	–
Crude ash (%)	6.60	7
Ca (%)	0.85	–
P (%)	0.65	–
Na (%)	0.50	0.41
Mg (%)	0.10	–
Lysine (%)	1.70	–

“–” indicates not reported or not available.

**Figure 1:**
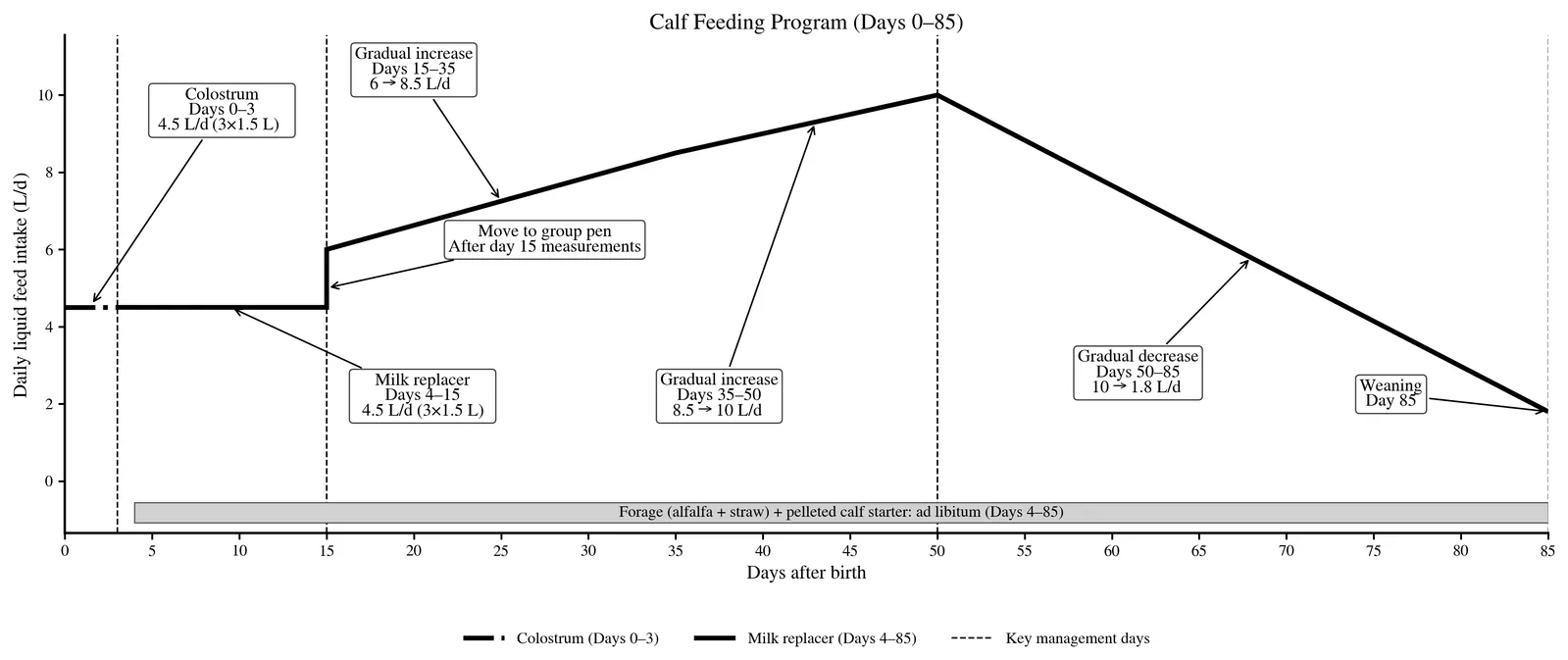
Milk-feeding programme applied to the experimental calves during the study period.

### 2.2 Body weight (BW) measurement, hair sample collection, and hair cortisol analysis

Throughout the experimental period, body weight (BW) was recorded using an electronic scale for all calves on seven occasions: at birth and on days 3, 15, 30, 45, 60, and 85 (weaning weight). At the beginning of the study, a subset of 14 calves was randomly selected for hair cortisol analysis, comprising 7 calves from each housing treatment. All 14 selected calves completed the 85 d follow-up. Hair sampling was initially planned to be performed at birth (within the first 24 h postpartum), on day 15, and on day 85 in these 14 calves. However, because hair regrowth was insufficient by day 15, hair samples were collected only at birth and on day 85 (weaning). Hair samples were obtained using the shave–reshave method from a 4cm×4cm area on the right shoulder region of each calf. At birth, the area was shaved as close to the skin as possible using a clipper, and, on day 85, the regrown hair was collected by reshaving the same area. All samples were placed in sealed plastic bags, transported to the laboratory, and stored at room temperature, protected from light and moisture until analysis.

Hair cortisol extraction was performed according to the procedure described by Ghassemi Nejad et al. (2020). Briefly, 250 mg of hair was washed three times with 5 mL of 96 % isopropanol and air-dried at room temperature for 1 week. After drying, 50 mg of hair was cut into very small fragments ( <1mm ) using surgical scissors and transferred into 2 mL microcentrifuge tubes. Methanol (1 mL ) was added, and tubes were shaken at 30 rpm for 24 h . Samples were then centrifuged for 30 s , and approximately 0.6 mL of the supernatant was transferred to a new tube and evaporated at 38 °C until completely dry. The dried residue was reconstituted in 0.4 mL phosphate-buffered saline (PBS; pH 7.5) and vortexed.

HCCs were quantified using a competitive ELISA kit (Cortisol ELISA Kit; Cayman Chemical, Ann Arbor, MI, USA; item no. 500360) according to the manufacturer's instructions. Absorbance was read at 405–420 nm , and concentrations were calculated from a four-parameter logistic standard curve. The assay working range was 6.6–4000 $\mathrm{pg}\;{\mathrm{mL}}^{-1}$ , with a reported sensitivity (80 % B/B0) of approximately 35 $\mathrm{pg}\;{\mathrm{mL}}^{-1}$ and a lower limit of detection (LLOD) of 26 $\mathrm{pg}\;{\mathrm{mL}}^{-1}$ . Manufacturer-reported intra- and inter-assay coefficients of variation ranged from 5.1 % to 13.4 % and from 6.7 % to 25.8 %, respectively, across the usable range of the standard curve. Antibody cross-reactivity was 15 % for dexamethasone, whereas cross-reactivity for most structurally related steroids was <5% . All samples were analysed in duplicate using a microplate reader (BioTek Epoch, USA), and washing steps were performed using a microplate washer (RAYTO, China).

### 2.3 Statistical analysis

All statistical analyses were performed using R software version 4.4.1 (R Core Team, 2024). Time was treated as a categorical variable and comprised days 0, 3, 15, 30, 45, 60, and 85 for body weight (BW); the interval days 0–3, 3–15, 15–30, 30–45, 45–60, and 60–85 for average daily gain (ADG); and days 0 and 85 for hair cortisol concentration (HCC).

The primary analyses presented in Table 2 were based on calves that completed the entire 85 d follow-up. This complete-case population comprised 20 calves (individual housing, n=11 ; pair housing, n=9 ). BW and ADG were analysed using linear mixed-effect models fitted by restricted maximum likelihood (REML) using the “lme4” package (Bates et al., 2015). Housing treatment (individual vs. pair), time (sampling day or period), the treatment × time interaction, and calving ease (normal vs. dystocia) were included as fixed effects. Calf ID was included as a random effect to account for repeated measurements within calves.

HCC was analysed using a generalised linear model with a Gaussian distribution and identity link. The HCC model included housing treatment, time, the treatment × time interaction, and calving ease as fixed effects. The HCC analysis comprised the 14 calves randomly selected at the beginning of the study, including 7 calves for each housing treatment. All 14 selected calves completed the 85 d follow-up.

To evaluate the robustness of the primary results and the potential influence of attrition, separate sensitivity analyses were conducted. For BW and ADG, these analyses incorporated all available repeated observations obtained from the 31 calves initially enrolled in the study, comprising 19 calves assigned to individual housing and 12 calves assigned to pair housing as six pairs. The sensitivity models included the same fixed effects as the primary models. Random intercepts for calf ID and initial housing-unit ID (pair–pen ID) were included to account for repeated observations within calves and clustering within the initial housing units. Each individually housed calf had a unique housing-unit ID, whereas the two calves initially housed together shared the same housing-unit ID.

HCC was additionally reanalysed using a mixed-effect model that included calf ID and initial housing-unit ID as random effects. No values were imputed after a calf left the study. Therefore, the sensitivity analyses used all observations available before death, with the number of observations being allowed to vary among measurement times. The sensitivity analyses were used to assess the robustness of the findings and did not replace the primary estimates presented in Table 2.

**Table 2:** Least-squares means ± standard errors (LSM ± SE) for BW (kg), ADG ( $\mathrm{kg}\;d^{-1}$ ), and HCC ( $\mathrm{pg}\;{\mathrm{mL}}^{-1}$ ) in the primary complete-case population.*

Time	Treatment		
	Individual ( n=11 )	Pair ( n=9 )	Overall ( n=20 )
BW (kg)			
Birth (day 0)	34.77±2.51	34.82±2.59	34.80±1.86 ^a^
Day 3	36.85±2.51	36.40±2.65	36.62±1.86 ^a,b^
Day 15	35.38±2.51	36.38±2.65	35.88±1.86 ^a^
Day 30	43.99±2.51	40.21±2.65	42.10±1.86 ^b^
Day 45	55.84±2.51	53.53±2.65	54.68±1.86 ^c^
Day 60	69.96±2.51	69.01±2.65	69.48±1.86 ^d^
Day 85 (weaning)	90.74±2.51	87.17±2.65	88.95±1.86 ^e^
ADG ( $\mathrm{kg}\;d^{-1}$ )			
Birth (day 0)–day 3	0.77±0.09 ^C,D^	0.59±0.10 ^B,C^	0.68±0.07 ^c^
Day 3–15	-0.10±0.09 ^A^	0.02±0.10 ^A^	-0.04±0.07 ^a^
Day 15–30	0.58±0.09 ^B,C^	0.26±0.10 ^A,B^	0.42±0.07 ^b^
Day 30–45	0.82±0.09 ^C,D^	0.92±0.10 ^C,D^	0.87±0.07 ^c,d^
Day 45–60	0.92±0.09 ^C,D^	1.02±0.10 ^D^	0.97±0.07 ^d^
Day 60–85 (weaning)	0.85±0.09 ^C,D^	0.75±0.10 ^C,D^	0.80±0.07 ^c,d^
HCC ( $\mathrm{pg}\;{\mathrm{mL}}^{-1}$ )			
Time	Individual ( n=7 )	Pair ( n=7 )	Overall ( n=14 )
Birth (day 0)	1152.60±112.35	1073.16±93.43	1112.88±79.97 ^b^
Day 85 (weaning)	570.37±112.35	640.31±93.43	605.34±79.97 ^a^

^*^ The study initially included 31 calves (individual housing, n=19 ; pair housing, n=12 ; six pairs). The BW and ADG estimates were based on 20 calves that completed the 85 d follow-up (individual housing, n=11 ; pair housing, n=9 ). The HCC estimates were based on 14 calves randomly selected at the beginning of the study ( n=7 per housing treatment). Sensitivity analyses incorporating all available observations are presented in Table S1 in the Supplement.^a–e^ Superscripts following the SE indicate differences among time points within the same parameter ( P<0.05 ).^A–D^ Superscripts following the SE indicate differences among time points within the same treatment ( P<0.05 ).

The significance of the fixed effects was evaluated using Wald $\chi^{2}$ tests. When a significant treatment × time interaction was detected, pairwise comparisons of estimated marginal means (least-squares means) were performed between housing treatments within each measurement time and among measurement times within each housing treatment. Tukey's adjustment was applied to account for multiple comparisons. The primary model results are presented as least-squares means and standard errors (LSM ± SE). Compact-letter displays for significant pairwise comparisons were generated using the algorithm described by Piepho (2004), as implemented in the “cld” function in R. Statistical significance was declared at α=0.05 .

## 3 Results

Of the 31 calves initially enrolled in the study, 20 completed the 85 d follow-up and were included in the primary complete-case analysis. The primary analysis population comprised 11 calves assigned to individual housing and 9 calves assigned to pair housing. A total of 11 calves died before completion of the follow-up, including 8 of the 19 calves assigned to individual housing and 3 of the 12 calves assigned to pair housing. The numbers of calves contributing BW measurements on days 0, 3, 15, 30, 45, 60, and 85 were 31, 30, 26, 21, 21, 20, and 20, respectively. The corresponding numbers contributing ADG observations during days 0–3, 3–15, 15–30, 30–45, 45–60, and 60–85 were 30, 26, 21, 21, 20, and 20, respectively. All 14 calves selected for HCC analysis completed the follow-up and contributed measurements at both birth and day 85.

Least-squares means (LSMs) and standard errors (SEs) for BW, ADG, and HCC in the primary analysis population are presented in Table 2. Housing treatment (individual vs. pair) had no significant main effect on BW or HCC ( P>0.05 ), whereas time affected both variables significantly ( P<0.05 ; Figs. 2 and 4).

**Figure 2:**
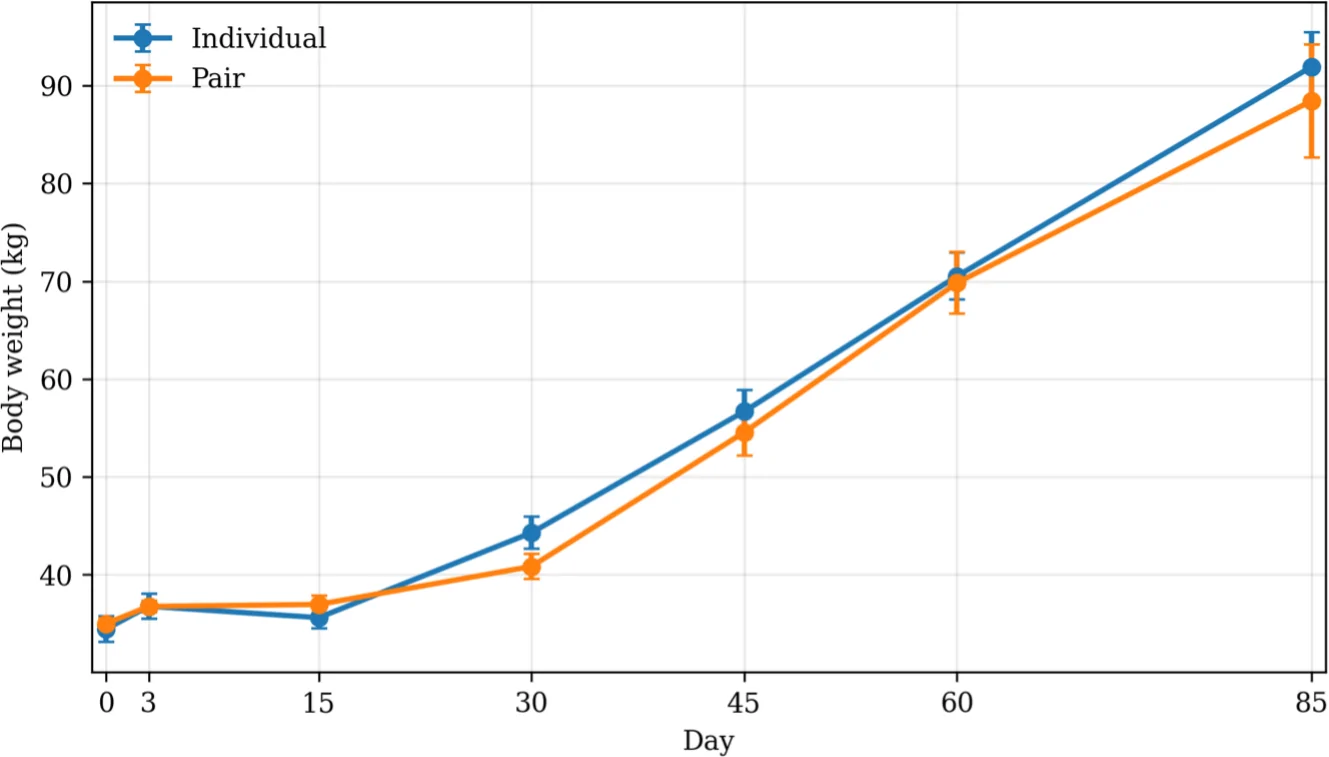
Changes in body weight (BW) of individually and pair-housed calves throughout the experimental period.

The treatment × time interaction was significant for ADG ( P<0.05 ; Fig. 3). During days 15–30, ADG was significantly higher in calves initially housed individually than in calves initially housed in pairs ( P<0.05 ). The highest ADG values were observed between days 45 and 60, whereas the lowest values occurred between days 3 and 15 in both groups.

**Figure 3:**
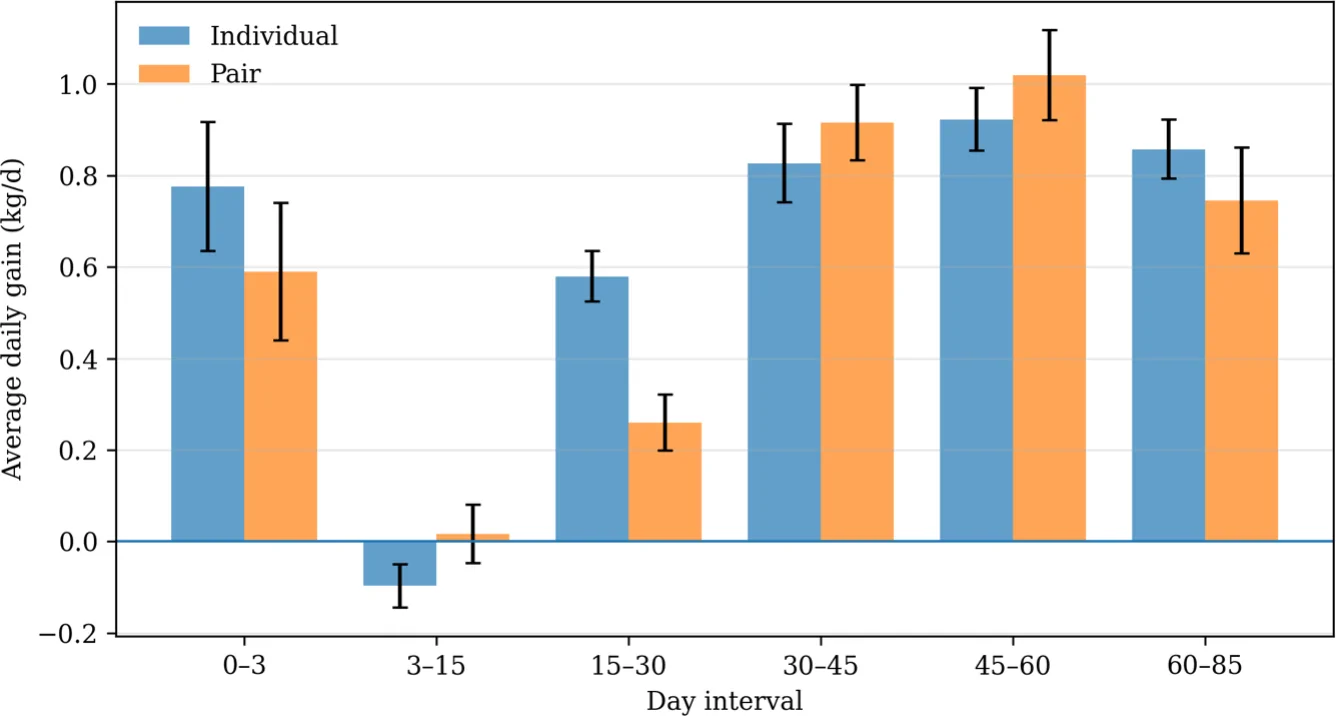
Changes in average daily gain (ADG) of individually and pair-housed calves throughout the experimental period.

Housing treatment had no significant main effect on HCC ( P>0.05 ), whereas HCCs at birth were significantly higher than those measured on day 85 ( P<0.05 ; Fig. 4). In addition, calving ease (normal vs. dystocia) had no significant effect on the evaluated traits ( P>0.05 ).

**Figure 4:**
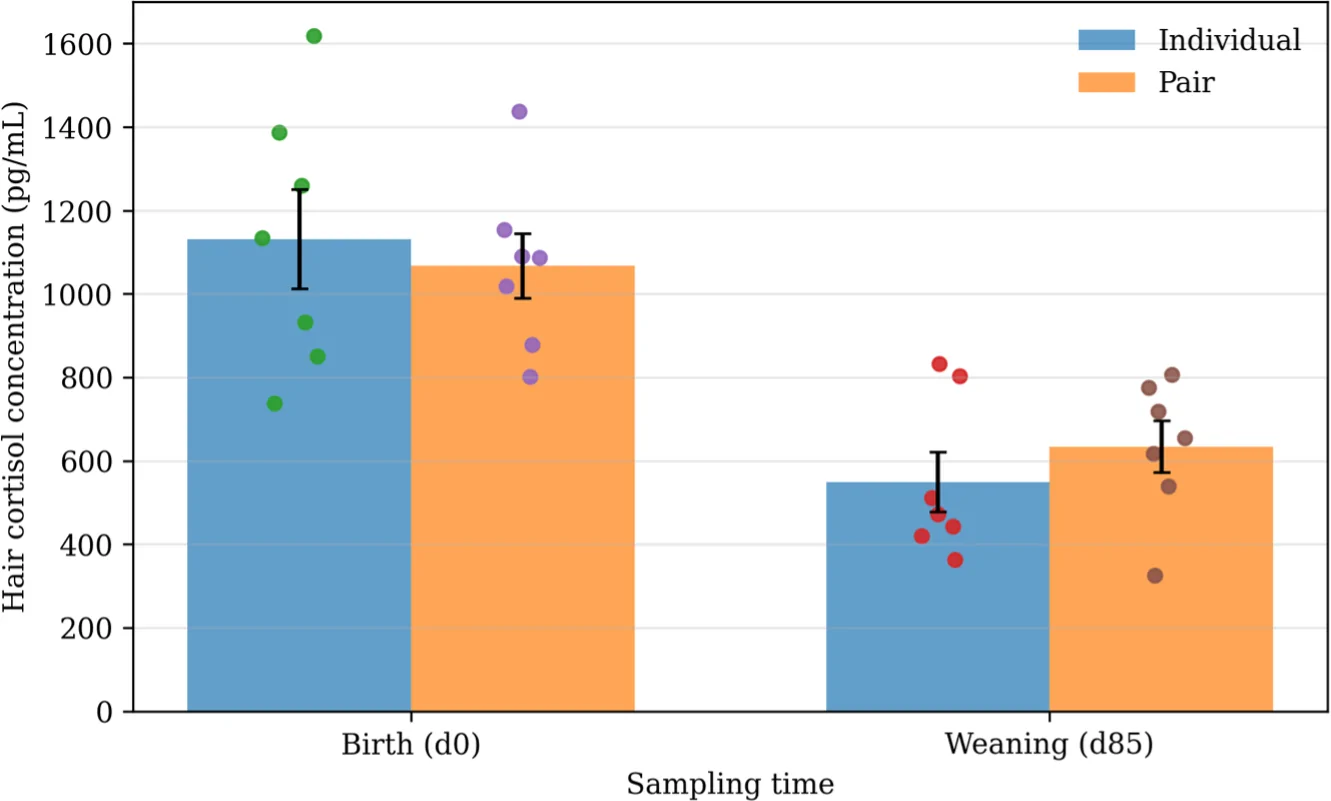
Changes in hair cortisol concentrations of individually and pair-housed calves throughout the experimental period.

The sensitivity analyses produced the same substantive interpretation as the primary analyses (Table S1). When all available BW observations from the 31 initially enrolled calves were analysed with calf ID and initial housing-unit ID included as random effects, the treatment × time interaction remained nonsignificant (Wald $\chi^{2}=4.253$ , df=6 , P=0.642 ). Similarly, the treatment × time interaction for ADG remained significant in the all-available-observations analysis (Wald $\chi^{2}=11.282$ , df=5 , P=0.046 ). The cluster-adjusted sensitivity analysis of HCC also showed no significant treatment × time interaction (Wald $\chi^{2}=0.739$ , df=1 , P=0.390 ). Accordingly, accounting for incomplete follow-up in the BW and ADG analyses and for clustering within the initial housing units did not alter the statistical interpretation of the primary findings.

## 4 Discussion

In the present study, LSMs for birth weight were 34.77 and 34.82 kg for individually and pair-housed calves, respectively, whereas LSMs for weaning weight were 90.74 and 87.17 kg , respectively (Table 2). Overall, we did not find statistical support for a main effect of individual versus pair housing during the first 15 d of the milk-feeding period on pre-weaning BW. Importantly, sensitivity analyses incorporating all available observations from the 31 initially enrolled calves and accounting for calf ID and initial housing-unit ID produced the same substantive interpretation as the primary complete-case analyses (Table S1 in the Supplement). Similar outcomes have been reported in previous studies (Chua et al., 2002; Jensen and Larsen, 2014; Jensen et al., 2015; Bolt et al., 2017; Aghaei et al., 2026). In contrast, other studies have suggested that pair housing may positively influence performance outcomes (de Paula Vieira et al., 2010; Liu et al., 2020; Knauer et al., 2021; Bonney-King et al., 2025).

We did not find statistical support for a main effect of housing treatment on BW, whereas the treatment × time interaction was significant for ADG. After transfer to group housing, ADG during days 15–30 was significantly higher in calves initially housed individually than in calves initially housed in pairs. This period-specific ADG difference may be related to behavioural adaptation and changes in feeding behaviour after regrouping. During this period, all calves received the same milk replacer allowance, while a hay–alfalfa mixture and calf starter were offered ad libitum. Although solid-feed intake was not measured in the present study, previous research indicates that pair housing may increase concentrate intake. Jensen et al. (2015) reported greater concentrate intake in pair-housed calves up to 6 weeks of age. Similarly, Liu et al. (2020) observed higher feed intake in pair-housed calves from week 3 to week 9, which they attributed to social learning. Aghaei et al. (2026) also reported higher total solid-feed intake and calf starter consumption in pair-housed calves from 4 to 65 d of age compared with individually housed calves. In agreement, de Paula Vieira et al. (2010) reported that pair-housed calves consumed more concentrate before weaning than individually housed calves, likely due to socially facilitated feeding.

Mean ADG from birth to weaning was 0.64 $\mathrm{kg}\;d^{-1}$ in individually housed calves and 0.59 $\mathrm{kg}\;d^{-1}$ in pair-housed calves. These values were comparable to those reported by Jensen and Larsen (2014) and Broucek et al. (2020). When ADG was examined across periods, the highest values were observed between days 45 and 60 in both groups, whereas the lowest values occurred between days 3 and 15. The period between days 3 and 15 corresponds to the transition from colostrum feeding to milk replacer and represents the most pronounced change in the feeding programme. Notably, during this period, the individually housed group exhibited negative mean ADG values, indicating a temporary reduction in body weight. In contrast, calves housed in pairs generally maintained body weight during the same period. Although both groups received the same amount of milk replacer, the differences in ADG during this interval may have been influenced by stress-related responses. Previous studies have suggested that housing calves in pairs or groups may facilitate adaptation to novel environments and management changes (de Paula Vieira et al., 2010; Bolt et al., 2017).

In the present study, calves were moved from the individual or pair pens to the group pen, and, during the period from day 15 to day 30, ADG was significantly higher in calves initially housed individually than in calves initially housed in pairs. Although behavioural monitoring was not performed during this period, the lower ADG observed in pair-housed calves might be related to the stress of being with multiple calves instead of their pen-mate and attempts to re-establish social contact after transfer to group housing. However, because behavioural responses, individual feed intake, and social interactions were not measured, this explanation remains speculative. Consistently with this performance pattern, Liu et al. (2020) reported lower ADG in pair-housed calves following transfer to group pens. From day 30 to day 60, mean ADG increased steadily in both groups and reached the highest values between days 45 and 60. Thereafter, mean ADG decreased continuously until weaning. The declining trend in ADG observed in both groups may be associated with the reduction in milk replacer allowance during the same period. In the current study, milk replacer was gradually reduced from day 50 onwards to prepare calves for weaning. Similarly, Jensen et al. (2015) reported a decrease in ADG when the daily milk allowance was reduced from 9 to 5 L . Overall, this pattern is consistent with the expected growth response to step-down milk-feeding strategies during the pre-weaning period.

In this study, housing treatment had no significant main effect on HCC, whereas HCCs at birth were significantly higher than those measured at day 85. To our knowledge, limited evidence is available for direct comparison of HCCs at weaning in calves housed individually versus in pairs. However, in agreement with our findings, Knauer et al. (2021) reported no effect of individual versus pair housing on HCCs measured at 16 weeks of age after weaning.

Hair cortisol measured at birth primarily reflects prenatal cortisol exposure rather than postnatal housing conditions. HCCs measured at day 85 represent cumulative cortisol deposition during the milk-feeding period. Several factors may influence neonatal HCCs; however, these were not evaluated in the present study because calves were born as singletons from primiparous dams, were of the same sex, and had similar birth weights. The higher HCCs at birth compared with those at weaning are consistent with the findings of Sano et al. (2023), who reported higher HCCs at birth than at day 30 postpartum. Furthermore, del Rosario González-de-la-Vara et al. (2011) reported that calves had higher HCCs than adult cows, and Heimbürge et al. (2020) found that newborn calves had higher HCCs than 6-month-old, 18-month-old, and adult cows.

Several limitations should be considered when interpreting these findings. Although farm health records and veterinary assessments indicated that the causes of death were unrelated to the experimental housing treatments, the primary analyses were based on the 20 calves that completed the 85 d follow-up, which may have introduced attrition or survivor bias. Sensitivity analyses using all available observations produced the same statistical interpretation; nevertheless, the limited sample size, differential attrition between housing treatments, and the small number of initial pairs reduce statistical precision and limit the generalisability of the findings. In addition, individually and pair-housed calves were kept in pens of the same dimensions ( 1.00m×1.95m ), resulting in space allowances of 1.95 $m^{2}$ per calf in individual housing and approximately 0.98 $m^{2}$ per calf in pair housing. Thus, housing treatment was confounded by space allowance or stocking density, and the observed findings cannot be attributed exclusively to social contact. These results should therefore be interpreted cautiously and confirmed in larger studies in which social contact and space allowance can be evaluated separately.

## 5 Conclusion

We did not find statistical support for a main effect of individual versus pair housing during the first 15 d on pre-weaning BW or HCC. However, the treatment × time interaction was significant for ADG, with higher ADG during days 15–30 in calves initially housed individually after transfer to group housing. Sensitivity analyses incorporating all available observations supported the substantive interpretation of the primary complete-case analyses. Nevertheless, the findings should be interpreted cautiously because of the limited sample size, attrition during follow-up, the small number of initial pairs, and the confounding of housing treatment with space allowance per calf.

## Supplement

10.5194/aab-69-643-2026-supplement The supplement related to this article is available online at https://doi.org/10.5194/aab-69-643-2026-supplement.

## Data Availability

The data supporting the findings of this study are not publicly available due to a confidentiality undertaking given to the private farm where the study was conducted. The data may be made available by the corresponding author upon reasonable request, subject to prior permission from the farm owner.
