# Supplementary material for: Effects of individual versus pair housing during the first 15 d on pre-weaning growth performance and hair cortisol concentrations in dairy calves
Source: Arch Anim Breed. 2026 Nov 9;69(4):643–51. doi: 10.5194/aab-69-643-2026 (PMC13752382; doi:10.5194/aab-69-643-2026)
Supplement: Supplementary file 1 [file aab-69-643-2026-supplement.pdf]

Supplement of Arch. Anim. Breed., 69, 643–651, 2026
https://doi.org/10.5194/aab-69-643-2026-supplement
© Author(s) 2026. CC BY 4.0 License.

Open Access
Archives Animal Breeding

Supplement of

Effects of individual versus pair housing during the first 15 d on pre-weaning growth performance and hair cortisol concentrations in dairy calves

Şevval Alsaç et al.

Correspondence to: Erkan Pehlivan (pehlivan@agri.ankara.edu.tr)

The copyright of individual parts of the supplement might differ from the article licence.

Table S1. Comparison of the primary complete-case analyses and sensitivity analyses for body weight, average daily gain, and hair cortisol concentration.

Outcome	*Analysis set*	*Calves, I/P (total)*	*Repeated observations, n*	*Random effects*	*Wald χ²*	*df*	*P value*
BW	Primary complete-case	11/9 (20)	140	Calf ID	3.694	6	0.718
BW	All-available sensitivity	19/12 (31)	169	Calf ID + initial housing-unit ID	4.253	6	0.642
ADG	Primary complete-case	11/9 (20)	120	Calf ID	11.979	5	**0.035**
ADG	All-available sensitivity	19/12 (31)	138	Calf ID + initial housing-unit ID	11.282	5	**0.046**
HCC	Primary analysis	7/7 (14)	28	None (fixed-effects model)	0.739	1	0.390
HCC	Cluster-adjusted sensitivity	7/7 (14)	28	Calf ID + initial housing-unit ID	0.739	1	0.390

BW: body weight, ADG: average daily gain, HCC: hair cortisol concentration, I: individual housing, P: pair housing.

For BW and ADG, the all-available sensitivity analyses included every observed measurement recorded before death among the 31 initially enrolled calves.

For HCC, the same 14 randomly selected calves contributed 28 observations because all selected calves completed follow-up.

Initial housing-unit ID was unique for each individually housed calf and shared by the two calves initially housed as a pair.

Wald χ^2 statistics and P values refer to the joint test of the housing treatment × time interaction. P values < 0.05 are shown in bold.
